# 脾切除治疗普通变异型免疫缺陷病合并血细胞减少一例报告并文献复习

**DOI:** 10.3760/cma.j.issn.0253-2727.2021.10.009

**Published:** 2021-10

**Authors:** 翠云 曲, 葳 刘, 荣凤 付, 云飞 陈, 晓帆 刘, 磊 张, 仁池 杨, 峰 薛

**Affiliations:** 中国医学科学院血液病医院（中国医学科学院血液学研究所），实验血液学国家重点实验室，国家血液系统疾病临床医学研究中心，天津 300020 State Key Laboratory of Experimental Hematology, National Clinical Research Center for Blood Diseases, Institute of Hematology & Blood Diseases Hospital, Chinese Academy of Medical Sciences & Peking Union Medical College, Tianjin 300020, China

**Keywords:** 普通变异型免疫缺陷病, 血细胞减少, 脾切除, Common variable immunodeficiency, Autoimmune cytopenia, Splenectomy

## Abstract

**目的:**

提高对脾切除术治疗普通变异型免疫缺陷病合并血细胞减少的认识。

**方法:**

报告1例脾切除治疗普通变异型免疫缺陷病合并血细胞减少病例并进行文献复习。

**结果:**

患者，女，16岁，因发现血小板减少8年入院。患者于青少年期发病，以反复血小板减少伴白细胞减少为主，感染症状较轻，脾肿大，血浆免疫球蛋白水平明显减低，诊断为普通变异型免疫缺陷病。给予足量静脉注射免疫球蛋白替代治疗后行脾切除术，脾脏病理示脾功能亢进，术后血细胞恢复正常水平。

**结论:**

普通变异型免疫缺陷病临床表现多样，可合并脾肿大、血细胞减少，在足量静脉注射免疫球蛋白替代治疗的前提下，对于反复血细胞减少的脾肿大患者，脾切除是一种安全有效的治疗方式。

## Abstract (en)

**Objective:**

To improve the understanding of splenectomy for treating common variable immunodeficiency complicated with cytopenia.

**Methods:**

A case of common variable immunodeficiency complicated with cytopenia was reported, and the literature was reviewed.

**Results:**

The patient, female, 16years old, was hospitalized for eight years due to thrombocytopenia; she manifested recurrent thrombocytopenia with leukopenia since adolescence. The patient was diagnosed with common variable immunodeficiency with repeated mild infections, splenomegaly, and significantly reduced plasma immunoglobulin levels. Additionally, splenectomy was performed with adequate immunoglobulin replacement therapy, and the pathology confirmed hypersplenism; her blood cell level returned to normal after surgery.

**Conclusion:**

Common variable immunodeficiency has various clinical manifestations and can be complicated with cytopenia. Under the premise of adequate immunoglobulin replacement therapy, splenectomy is a safe and effective treatment for common variable immunodeficiency in patients with recurrent cytopenia.

普通变异型免疫缺陷病（common variable immunodeficiency, CVID）是一种常见的原发性免疫缺陷病，以B细胞分化成熟缺陷、抗体产生受损和低免疫球蛋白血症为主要特点，患病率1/100 000～10/100 000[1]–[2]。该病临床表现多样，除反复呼吸道、消化道感染外，还有非感染性表现，如自身免疫病、肉芽肿病、淋巴增殖性疾病等[3]–[5]。自身免疫病中最常见的是自身免疫性血细胞减少，包括原发免疫性血小板减少症（ITP）和自身免疫性溶血性贫血（AIHA）等[3],[6]–[7]。ITP的存在给CVID的治疗带来挑战。虽然近年来治疗方案不断更新，对于慢性ITP，尽管术后感染风险增加，脾切除仍然是一种有效的治疗方式。对于CVID并发的ITP，脾切除可能会加重潜在的免疫缺陷，增加感染风险。国内鲜有脾切除治疗CVID合并ITP的报道，我们报告国内首例因持续血小板减少、白细胞减少、巨脾行脾切除的CVID病例，并对脾切除在CVID中的应用进行文献复习。

## 病例资料

患者，女，16岁，蒙古族，因发现血小板减少8年入院。患者8年前因感冒、磕碰后皮肤瘀斑就诊于当地医院，查血常规发现血小板计数（20～30）×10^9^/L，诊断为“ITP”，给予“激素、静脉注射免疫球蛋白（IVIG）”治疗，血小板升至正常水平，后未再复查，无出血症状。3年前再次因感冒、皮肤瘀斑在当地就诊，血常规示PLT 23×10^9^/L，WBC、HGB正常，拟诊为“ITP、骨髓增生异常综合征（MDS）不能除外”，给予“曲安西龙、达那唑”治疗，血小板计数升至正常水平。1年前无明显诱因出现双下肢皮肤瘀点在当地就诊，检查发现TP53突变（突变位点E11Q，突变率50.23％）、TET2突变（突变位点F868L，突变率50.23％），考虑“ITP，MDS不能除外”。给予“输注血小板、IVIG、重组人血小板生成素”治疗，血小板恢复正常。出院后应用“曲安西龙、达那唑”治疗。近2个月血小板水平逐渐下降，伴磕碰后皮肤瘀斑而就诊于我院。否认家族遗传病史。查体：体温36.3 °C，脉率86次/min，呼吸21次/min，血压93/61 mmHg（1 mmHg = 0.133 kPa）。周身皮肤无皮疹、黄染，下肢皮肤散在瘀斑，无贫血貌。双肺呼吸音清，未闻及干湿性啰音。肝肋缘下未触及，脾甲乙线7 cm。血常规：WBC 3.53×10^9^/L，中性粒细胞1.33×10^9^/L，RBC 4.61×10^12^/L，HGB 139 g/L，PLT 35×10^9^/L。肝功能：总蛋白59.3 g/L，白蛋白42.3 g/L，球蛋白17 g/L，丙氨酸转氨酶51.1 U/L，天冬氨酸转氨酶51.6 U/L。免疫球蛋白定量：IgA 0.14 g/L（参考值0.82～4.53 g/L），IgG 3.99 g/L（参考值7.51～15.6 g/L），IgM 0.12 g/L（参考值0.46～3.04 g/L）。抗核抗体、ENA抗体谱、血小板膜糖蛋白抗体检测阴性。HIV抗体阴性，EB病毒抗体、巨细胞病毒抗体均阴性。淋巴细胞亚群：淋巴细胞占有核细胞的59％，CD19^+^ CD20^+^ B细胞占淋巴细胞4.9％。M蛋白鉴定、肿瘤标志物检测阴性。全外显子组测序未发现疾病相关突变。骨髓穿刺涂片：粒系比例减低，红系比例增高，巨核系增生。骨髓病理：骨髓增生大致正常。骨髓免疫分型（MDS/MPN）未见异常。免疫组织化学染色示正常巨核细胞27个。胸部CT：两肺间质纹理增多，多发斑片影、小结节样影及小树芽，部分实变，考虑感染性病变，两侧胸膜增厚，脾脏增大。腹部彩超：肝脏大小形态正常；脾脏重度大，长18.9 cm，厚4.9 cm，肋缘下6.9 cm×3.4 cm。临床诊断为“ITP、CVID、白细胞减少、脾大、脾功能亢进”。住院期间给予大剂量IVIG及艾曲波帕（50 mg单剂）治疗，出院时血常规：WBC 1.97×10^9^/L，HGB 127 g/L，PLT 25×10^9^/L，中性粒细胞0.82×10^9^/L。出院后应用IVIG替代治疗（0.4 g/kg，每月1次），同时应用艾曲波帕（由50 mg/d逐渐调整为间隔2～3 d 50 mg）。艾曲波帕减量过程中，血小板计数最低降至17×10^9^/L，白细胞计数最低2.44×10^9^/L。因脾脏重度大伴血小板、白细胞水平持续减低，拟行腹腔镜脾切除术。术前半个月开始应用艾曲波帕50 mg/d，术前2 d输注IVIG 0.4 g/kg，术前1 d血小板计数升至51×10^9^/L。手术当天输注血小板2 U，手术过程顺利。术后3 d血小板计数达峰值291×10^9^/L后逐渐下降，术后4周复查PLT 50×10^9^/L，术后6周逐渐升至正常水平；术后白细胞计数持续处于正常水平。术后规律应用IVIG替代治疗，术后8个月血常规示PLT 300×10^9^/L，未发生感染性疾病。脾脏术后病理：脾窦增生，淋巴组织减少，符合脾功能亢进。

## 讨论及文献复习

CVID为最常见的原发性免疫缺陷病，其临床特点是血浆IgG伴IgA/IgM减少，造成反复呼吸道、消化道感染，同时可能伴有自身免疫病、脾肿大等表现。CVID以散在分布为主，男女皆可发病，常在2岁后起病，诊断年龄有儿童期、30～40岁两个高峰[8]。临床表现多样、发病年龄不等，造成CVID诊断相对困难，通常有4～6年的诊断延迟[8]。

CVID患者临床表现多样，反复感染最为常见，以呼吸道和消化道为主。除感染之外，还有自身免疫病、肉芽肿病、淋巴增殖性疾病甚至恶性肿瘤等表现。CVID患者中，20％～30％会发生自身免疫病[9]，最常见的表现为自身免疫性血细胞减少，发生率为11％～18％，主要是ITP和AIHA，中性粒细胞减少偶有发生[4]–[5],[7]–[8]。在超过60％的患者中，血细胞减少的诊断早于CVID的诊断[10]。本例患者以反复严重血小板减少为主要表现，感染症状较轻，但血小板减少的发生与感染相关，与Pituch-Noworolska等[10]报道的病例类似。即便在已经确诊CVID并接受IVIG规律替代治疗的患者，感染也可能使患者发生急性血小板减少或慢性血小板减少急性加重，这时IVIG治疗仍然有效。CVID患者恶性肿瘤的发生率是普通人群的6倍，而ITP是CVID患者发生恶性肿瘤的危险因素[11]。

脾肿大是CVID的常见临床表现，25％～30％的患者有此症状，但也有脾肿大发生率高达66％的报道[5],[8]。脾肿大的患者更易发生自身免疫性血细胞减少，B细胞成熟或选择受损可能是原因之一[3]。脾肿大还与其他自身免疫病、肉芽肿病、肝脏疾病和门脉高压有关[8]。虽然目前尚没有明确脾肿大与血细胞减少的具体机制，但我们的患者脾脏重度大，且切脾后血小板和白细胞水平明显升高，说明二者之间具有相关性。

血浆免疫球蛋白水平减低和B细胞功能障碍是CVID最主要的实验室检查异常。CVID患者血浆IgG水平普遍降低，部分患者甚至低于检测下限[8]。有研究者认为IgA降低是CVID的一个重要特征[1]。IgM水平多降低，但也有部分患者正常。

CVID的发病机制目前尚不清楚。B细胞功能受损在CVID患者中很普遍。尽管CVID患者的B细胞总数可能正常，但B细胞成熟过程受损会导致其功能异常。基于B细胞表型的流式细胞术已经用来识别不同的患者亚群[7]。在CVID患者中，转换型记忆B细胞减少和CD21^low^ B细胞扩增与自身免疫和脾肿大相关[3]。一项欧洲多中心研究发现，大多数CVID患者的转换型记忆B细胞严重减少，这与脾肿大和肉芽肿病高风险相关[7]。CD21^low^ B细胞来源于经历慢性免疫刺激的成熟B细胞，在CVID患者中增加，在合并ITP的CVID患者中水平更高，表明其表征着一个特殊的B细胞亚群，可能代表感染和自身免疫之间的联系，而CD21^low^ B细胞扩增预示患者可能发生脾肿大[7],[9]。

通过基因组关联研究和基因组或外显子组测序技术，研究人员已经发现了导致CVID的若干基因缺陷，最常见的是涉及B细胞活化的基因。然而这些缺陷仅能解释不到15％的CVID患者，表明CVID并非简单的单基因遗传病，遗传、环境和免疫因素的融合可能是CVID发病的基础，复杂的病因也解释了CVID的不同临床表现和迟发性[3],[9],[12]。本例患者全外显子组测序未发现疾病相关突变，也侧面印证了这一点。通过全基因组测序有可能会发现新的致病基因。

CVID的诊断主要依据临床表现和免疫学检查。国内尚无CVID的诊断标准，目前多采用欧洲免疫缺陷协会（ESID）/全美免疫缺陷协作组（PAGID）的诊断标准[9]：IgG明显减低（至少低于相应年龄段平均值2个标准差），同时，IgA或者IgM明显减低，并需要满足以下标准：①发病年龄>2岁；②对疫苗反应差；③除外其他疾病。

在某些情况下，自身免疫的发现可能早于免疫缺陷，免疫抑制剂的使用可能会加重潜在的免疫功能缺陷[3],[10]。故而，CVID的早期诊断显得尤为重要。2020年版成人原发免疫性血小板减少症诊断与治疗中国指南[13]提出，对疑诊原发性ITP的患者，应用IVIG治疗前，应检测血清IgG、IgA、IgM水平，以鉴别CVID合并ITP。对难治性ITP儿童患者进行高通量二代测序和详尽的病史回顾，有益于没有任何有意义临床特点的患者的早期诊断，可以区分CVID合并ITP与原发性ITP患者。对疑似CVID的患者进行进一步的检查和随访，以早期诊断并及时治疗，避免严重感染等情况的发生[14]。Arays等[15]曾报道1例因ITP接受脾切除和利妥昔单抗治疗女性患者，因发生严重感染而确诊为CVID。说明CVID早期诊断并及时给予IVIG替代治疗的重要性。

目前，IVIG已经是CVID患者的标准治疗，推荐剂量为每月0.4～0.8 g/kg，谷浓度应达到7 g/L。谷浓度<4 g/L者临床结局较差，谷浓度较高者严重细菌感染发生率明显降低，谷浓度每升高0.1 g/L，肺炎发生率降低27％[8]。

尽管存在感染风险增加的可能性，糖皮质激素仍然是CVID合并ITP的一线治疗。大剂量IVIG可用于已经接受IVIG替代治疗的高出血风险患者[6]。二线治疗中，利妥昔单抗已被证明是CVID相关自身免疫性血细胞减少（尤其ITP）安全、有效的治疗方法[6],[16]。

皮下注射免疫球蛋白（fSCIg）安全性、有效性和耐受性良好，甚至可以控制住IVIG无效的ITP，还可减少泼尼松用量，提示fSCIg可能具有独特的免疫调节作用[17]。

在CVID患者，脾切除通常用于合并难治性血细胞减少患者的治疗和疑诊淋巴瘤患者的诊断，或因巨脾而行脾切除[18]。对于反复发生血细胞减少的CVID患者，脾切除是一种有效的治疗手段，甚至在一些利妥昔单抗治疗失败的患者。而且，脾切除没有加重CVID的死亡率，足够的IVIG替代治疗可保护患者避免严重感染的发生[16],[19]–[20]。本例患者起病初期糖皮质激素和IVIG治疗有效，但病情反复。确诊CVID后，应用艾曲波帕治疗效果欠佳，足量IVIG替代治疗后进行脾切除，术后血小板、白细胞水平均恢复正常。术后继续规律IVIG替代治疗，至今随访8个月，白细胞和血小板水平正常，未发生感染。

脾切除与循环中IgM型记忆B细胞减少和肠道分泌IgA的浆细胞缺失有关，而循环中IgM型记忆B细胞减少的CVID患者消化道分泌IgA的浆细胞频率减低，上皮细胞分泌型IgA分泌谱破坏[21]。CVID患者进行脾切除，可能会加重潜在的黏膜免疫缺陷，增加消化道感染风险。这是临床医生及患者需要注意的情况。

Furudoï等[22]对17例CVID患者的脾切除样本分析显示，脾脏白髓增生伴反应性滤泡而无肉芽肿的患者多为女性，CVID发病、诊断和脾切除时较年轻但延迟诊断时间较长；有更多的自身免疫病、肺部并发症和肝脏结节性增生；其IgA、IgG、IgM水平也较高。脾脏病理示肉芽肿，有或无白髓增生的患者多为男性，CVID发病、诊断和脾切除时年龄较大；有弥漫性肉芽肿疾病，但是感染、自身免疫等情况少见；免疫球蛋白水平较低。本例患者脾脏病理示脾窦增生，淋巴组织减少，故而考虑脾功能亢进在血细胞减少中发挥重要作用。

本例患者诊疗过程提示，对合并自身免疫病尤其自身免疫性血细胞减少的CVID患者，在规律IVIG替代治疗的前提下，应用糖皮质激素、利妥昔单抗相对安全；对于反复发生血细胞减少的患者，脾切除可能是一种疗效持久且相对安全的治疗方式。
